# Prevalence and coexistence of locomotive syndrome with reduced mobility and metabolic syndrome: a cross-sectional study of 35,059 Japanese adults

**DOI:** 10.1038/s41598-025-98288-2

**Published:** 2025-04-19

**Authors:** Chihiro Goto, Kohei Maruya, Yasuhiro Morita, Tomoyuki Arai, Satoshi Yamaguchi, Keiko Yamada, Masaru Moriyama, Hideaki Ishibashi, Ryo Nakagawa

**Affiliations:** 1 Division of Advanced Preventive Medicine, Chiba University, 1-8-1 Inohana, Chuo-ku, Chiba, 260-8670 Japan; 2 Department of Gastroenterology, Graduate School of Medicine, Chiba University, 1-8-1 Inohana, Chuo-ku, Chiba, 260-8670 Japan; 3 Omiya City Clinic, 1-7-5 Sakuragicho, Omiya-ku, Saitama City, Saitama 330-8669 Japan; 4 Department of Physical Therapy, School of Health Sciences, Japan University of Health Science, 2-555, Suga, Satte, Saitama 340-0145 Japan; 5 Department of Physical Therapy Faculty of Health and Medical Care, Saitama Medical University, 981, Kawakado, Iruma, Saitama 350-0436 Japan; 6 Graduate School of Global and Transdisciplinary Studies, Chiba University, 1-8-1 Inohana, Chuo-ku, Chiba, 260-8670 Japan; 7 Department of Liberal Arts, Faculty of Healthcare and Welfare, Saitama Prefectural University, 820 Sannomiya, Koshigaya-shi, Saitama 343-8540 Japan; 8 Department of Rehabilitation, The University of Tokyo Hospital, 7-3-1, Hongo, Bunkyo-ku, Tokyo, 113-0033 Japan; 9 Department of Orthopedic Surgery, INA Hospital, 5014-1, Komuro, Kitaadachi-Ina, Saitama 362-0806 Japan

**Keywords:** Disability, Locomotive syndrome, Metabolic syndrome, Health care, Medical research

## Abstract

Metabolic syndrome (MetS), marked by visceral obesity, hypertension, hyperglycemia, and dyslipidemia, elevates cardiovascular risk. Locomotive syndrome (LS), characterized by age-related mobility decline, often precedes frailty and sarcopenia. We investigated the prevalence and association between MetS and LS among Japanese middle-aged and older adults. This retrospective cross-sectional study was conducted using clinical records of 35,059 Japanese adults who underwent health check-ups between April 2021 and March 2022. MetS was diagnosed using the modified National Cholesterol Education Program Adult Treatment Panel III criteria, and LS was assessed using the LS risk test, which includes the stand-up test, two-step test, and the 25-question Geriatric Locomotive Function Scale. Among the participants, 2640 (7.5%) were diagnosed with MetS, and 5265 (15.0%) were classified as LS-positive. The prevalence of LS was significantly higher in individuals with MetS. The association between MetS and LS remained significant after adjusting for age and sex. We found that LS prevalence was higher in women than in men, though the primary association between MetS and LS remained consistent across both sexes. This large-scale real-world analysis emphasizes the need for integrating LS screening into routine check-ups to detect and manage MetS, aiding in the prevention of functional decline in aging populations.

**Supplementary Information:**

The online version contains supplementary material available at 10.1038/s41598-025-98288-2.

## Introduction

In a rapidly aging society, early preventive medical approaches are increasingly needed to curb the rise of age-related frailty^1–4^. Impaired physical characteristics of frail older individuals include decreased muscle strength, reduced walking speed, and increased fatigue^5–8^. Moreover, there is a correlation between metabolic syndrome (MetS) and locomotor disorders in older people, with each exacerbating the other^9,10^. MetS is a cluster of conditions that occur together, increasing the risk of cardiovascular diseases and stroke. MetS is often accompanied by a decrease in muscle mass^11–13^, suggesting a potential association between its onset and frailty. However, it remains unclear at what age these musculoskeletal disorders and MetS begin to overlap and what characteristics define their early manifestations.

Since 2000, the Japanese government has launched “Healthy Japan 21,” a comprehensive health promotion campaign aimed at improving the Japanese population’s well-being^14,15^. One of the primary objectives of this initiative is to reduce the prevalence of MetS and pre-MetS conditions. Consequently, MetS screening has been integrated into Japan’s comprehensive health check-up programs, with individuals identified under this category receiving targeted guidance^16,17^. Moreover, since 2015, in the second term of this health campaign, the prevention of locomotive syndrome (LS), which is defined by the Japanese Orthopedic Association (JOA) as the age-related deterioration of mobility mainly due to dysfunction of a locomotor system, has been incorporated^15,18,19^. Consequently, an increasing number of medical assessment institutes are educating patients about LS and incorporating LS screening into physical check-ups.

The LS risk test developed by the JOA is a simple evaluative tool incorporating assessments of the ability to stand from a sitting position, the distance covered in two steps, and responses to a 25-question survey regarding physical and living conditions^19,20^. This test is gradually being incorporated into private comprehensive health check-ups in Japan to simultaneously assess MetS and declining mobility. However, a realistic data analysis integrating both syndromes based on health check-up data has not yet been conducted.

In this study, we used real-world data from Japanese health check-up facilities to determine the age groups which have the highest prevalence of MetS and LS. We then analyzed data on obesity, blood pressure, blood glucose, and lipid metabolism by comparing individuals with and without LS at the age when the prevalence of LS begins to rise, thereby elucidating the impact of LS on MetS.

## Methods

### Study design and participants

This retrospective observational study was conducted by analyzing the clinical records of all patients who underwent “Ningen Dock,” a private health check-up service at the Omiya City Clinic, between April 1, 2021 and March 31, 2022^21^. Data from patients who did not fulfill any of the diagnostic criteria for MetS or who did not take a locomotion test were excluded from this analysis.

This study was conducted in compliance with the Declaration of Helsinki. The research protocol was reviewed and approved by the Ethics Committee of Saitama Medical University (Approval number: 957). Due to the retrospective nature of the study, the requirement for informed consent was waived by the ethics committee. Instead, an opt-out approach was used for patient data disclosure. All patient data were anonymized to ensure confidentiality and privacy.

### Measures

#### MetS

The modified National Cholesterol Education Program (NCEP) adult treatment panel-III (ATP-III) was used to screen for MetS^22^. The criteria for MetS in Asians was waist circumferences of 90 cm for men and 80 cm for women, as these thresholds have been shown to be more predictive of cardiovascular risks in Asian populations compared to the original NCEP ATP III criteria^23^. From the health check-up database questionnaire, items related to the presence of or treatment for antihypertensive, diabetes, and lipid metabolic disorders were extracted, while waist circumference, systolic and diastolic blood pressure, fasting blood sugar, triglycerides, and high-density lipoprotein cholesterol were extracted from the measured laboratory results to screen for MetS.

#### LS risk test

The LS risk test is a method recommended by the JOA to assess reduced mobility, mainly due to dysfunction of the locomotor system^18,19^. This evaluation consists of three distinct tests: a stand-up test, a two-step test, and an assessment based on the 25-question geriatric locomotive function scale (GLFS-25). The final LS classification was determined based on the lowest performance among the three tests to comprehensively assess locomotive dysfunction. The outcomes of each test were classified into four risk levels: Non-LS (normal mobility), LS stage 1 (LS_1: onset of reduced mobility), LS stage 2 (LS_2: progression in mobility decline), and LS stage 3 (LS_3: advanced stage with impaired social participation), as outlined in Supplementary Table S1. In this study, patients classified with LS Stages 1–3 were collectively grouped and referred to as the ‘LS’ group.

The tests were included as part of the private health check-up, and all participants received the results. The detailed criteria for determining LS classification based on each of the three tests are described below.

#### Stand-up test

The standing test is used to determine lower limb muscle strength. First, the participant attempts to stand up from a 40-cm high chair with both feet. If they are unable to stand up, they are immediately classified as LS_3. If they can stand up using both feet, they are then challenged to rise up from a 40-cm high chair with only one foot. If the person fails, they are urged to try to attempt standing up with both legs from chairs of progressively lower heights (30, 20, and 10 cm). The height at which a person can stand up from the lowest height determines their LS stage, and the following classifications is applied. If the person is unable to stand up using one leg from a 40-cm chair but is able to stand up using both legs from a 20-cm chair, they are classified as LS _1; if they cannot stand with both feet from a 20-cm chair but can rise with both feet from a 30-cm chair, it is LS_2; and if they cannot stand using both feet from a 30-cm chair, it is LS_3.

#### Two-step test

The two-step test evaluated walking abilities by measuring stride lengths. Participants were instructed to take the longest possible two steps forward from a starting line. The total stride length from the starting point to the final toe position was measured and then divided by the participant’s height for evaluation. Based on the calculated two-step value, a Non-LS rating was assigned if the value was ≥ 1.3, LS_1 was between 1.1 and < 1.3, LS_2 was between 0.9 and < 1.1, and LS_3 was < 0.9.

#### GLFS-25

The GLFS-25 is a comprehensive self-assessment tool for evaluating locomotive syndrome, and examines physical conditions such as pain, activities of daily living, and mental health over the past month as outlined in Supplementary Table S2. Participants answer a sequence of 25 questions covering aspects such as pain in various parts of the body (neck, back, legs, and any other), difficulties with activities of daily living (getting up, walking, and changing clothes), and concerns about social participation and mobility (fear of falling and avoiding social activities). Each question is scored based on the level of difficulty or discomfort experienced, ranging from ‘no difficulty or discomfort’ to ‘severe difficulty or discomfort.’ The cumulative score determines the overall level of risk of LS: < 7 points is classified as non-LS, 7–16 points is classified as LS_1, 16–24 points is classified as LS_2, and ≥ 24 points is classified as LS_3.

### Outcomes

The primary outcome was the distribution and overlap between locomotive disorders and MetS in middle-aged and older adults undergoing physical examinations. The secondary outcome was the clinical characteristics of the middle-aged population with locomotor disorders.

### Statistical analyses

Data are presented as means and standard deviation for continuous variables and numbers and proportions (%) for categorical variables. A significance threshold of *P* < 0.05 was used for all statistical tests, while a more stringent threshold of *P* < 0.001 was applied for multiple comparisons and logistic regression analyses to account for the large sample size. All statistical analyses were conducted in JMP 17.1.0 (JMP Statistical Discovery LLC, NC, USA). To investigate the association between MetS and LS, statistical analyses were conducted as follows: *t*-tests were employed to assess mean differences, while chi-square tests were utilized to compare proportions. Logistic analysis was conducted to identify factors associated with LS. Additionally, mean comparisons were subjected to the Dunnett test, and proportions of cases presenting with both MetS and LS were evaluated using the chi-square test.

## Results

### Clinical background of participants who underwent health screening

During the study period, a total of 38,374 individuals underwent health check-ups, of which 35,059 who were examined for LS and MetS were included in this study (Table 1). Among these 35,059 participants, 20,875 (58%) and 14,184 (48%) were men and women, respectively. The percentage of participants in the age groups < 39, 40–49, 50–59, 60–69, and 70 years were 14.4%, 35.8%, 32.7%, 14.4%, and 2.8%, respectively. Among the 5265 (15%) risk-positive participants with LS, 12.7, 1.6, and 0.7% were classified as LS_1, LS_2, and LS_3, respectively.

**Table 1:** Distribution of locomotive syndrome and metabolic syndrome among 35,059 Asian individuals who underwent health check-ups.

	Overall	Metabolic syndrome		*P*-value
		With	Without	
Variables	n = 35,059	n = 2640 (7.5)	n = 32,419 (92.5)	
Demographics (%)				
Men	20,875 (59.5)	1,919 (72.7)	18,956 (58.5)	†
Women	14,184 (40.5)	721 (27.3)	13,463 (41.5)	
Age (Mean ± SD)	50 ± 9.6	52.5 ± 9.0	49.8 ± 9.7	*
Years (%)				†
< 39	5,035 (14.4)	195 (7.4)	4,613 (14.9)	
40–49	12,538 (35.8)	808 (30.6)	11,730 (36.2)	
50–59	11,474 (32.7)	1,067 (40.4)	10,407 (32.1)	
60–69	5,048 (14.4)	468 (17.7)	4,580 (14.1)	
> 70	964 (2.8)	102 (3.9)	862 (2.7)	
Locomotive syndrome assessment test (%)				†
Non-LS	29,794 (85.0)	2,018 (76.4)	2,776 (85.7)	
LS_1	4,442 (12.7)	510 (19.3)	3,932 (12.1)	
LS_2	573 (1.6)	78 (3.0)	495 (1.5)	
LS_3	250 (0.7)	34 (1.3)	216 (0.7)	
Clinical data (mean ± SD)				
Waist circumference (cm)	80.2 ± 9.5	93.4 ± 8.9	79.2 ± 8.7	*
Systolic blood pressure (mmHg)	117.3 ± 15.5	133.8 ± 15.0	115.9 ± 14.7	*
Diastolic blood pressure (mmHg)	73.5 ± 11.9	85.4 ± 11.1	72.5 ± 11.5	*
Glucose (mg/dL)	98.8 ± 15.3	117.1 ± 27.3	97.3 ± 12.7	*
HDL (mg/dL)	61.4 ± 71.2	46.6 ± 11.3	62.6 ± 15.4	*
TG (mg/dL)	97.9 ± 71.2	196.4 ± 125.3	89.8 ± 57.9	*
Treatment (%)				
Hypertension	4,722 (13.5)	880 (33.3)	3,842 (11.9)	†
Type 2 diabetes	1,273 (3.6)	403 (15.3)	870 (2.7)	†
Hyperlipidemia	3,893 (11.1)	620 (23.5)	3,273 (10.1)	†

*SD* standard deviation, *LS* Locomotive syndrome, *HDL* high-density lipoprotein, *TG* triglycerides.

*Significant difference by *t*-test (*P* < 0.001). †Significant difference in chi-square test (*P* < 0.001).

The number of people diagnosed with MetS was 2640 (7.5%), among which there were significantly more men and older people with LS than those in the non-MetS group (all *P* < 0.001). Clinical data for each group are also shown in Table 1.

### Distribution of LS among the participants who underwent health check-ups

**Fig. 1:**
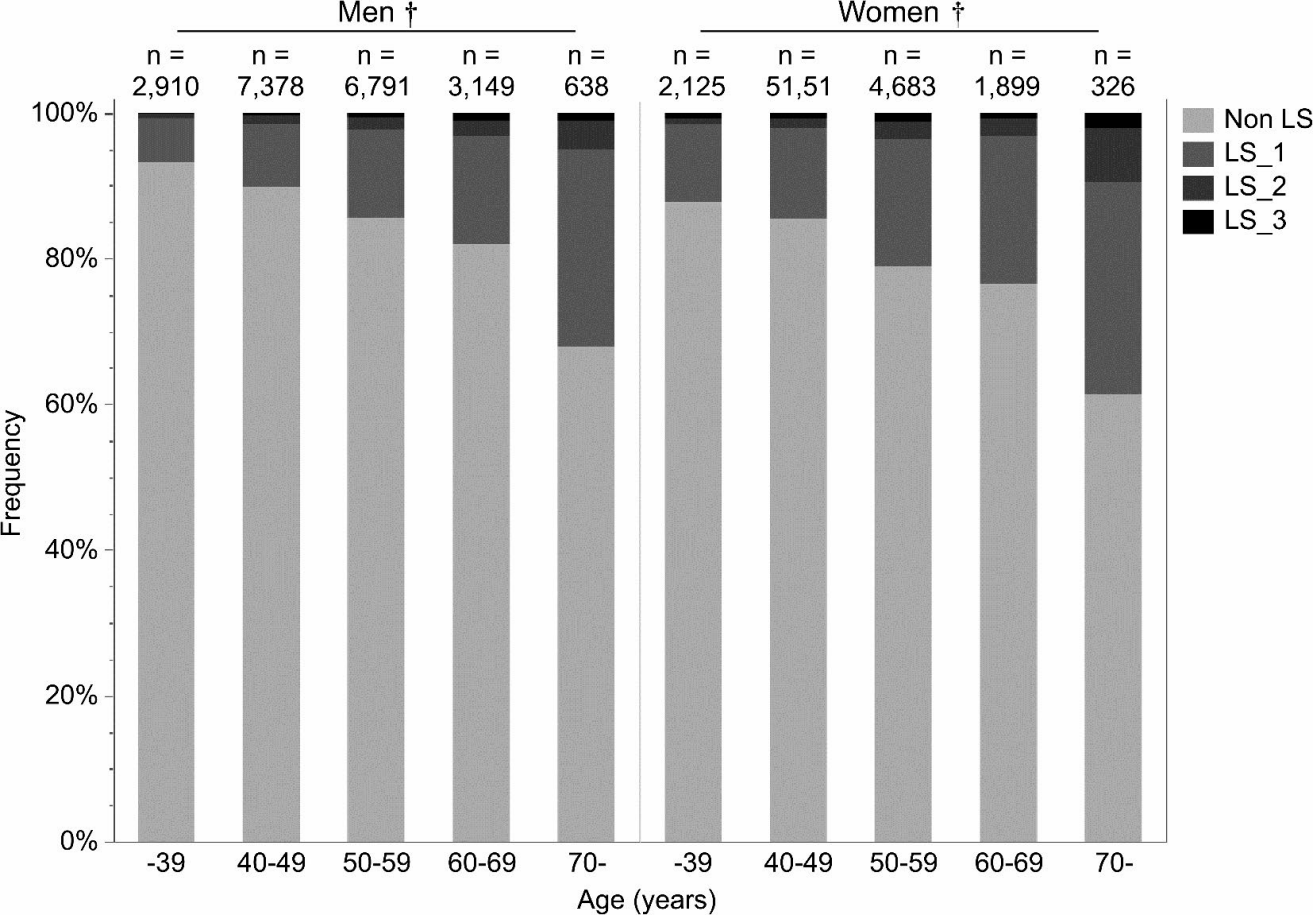
Age distribution of locomotive syndrome (LS) in men and women. This bar graph shows the proportions of the distribution of the outcomes of the locomotive syndrome risk test by sex and age. Proportions of the distribution of the outcomes of the Locomotive syndrome risk test by sex and age were compared using the chi-square test. †*P* < 0.001.

The number of participants with locomotor disorders was 2,699 men (7.7% of all men) and 2566 women (18.1% of all women) (*P* < 0.001). Analysis using the chi-square test showed that the proportion of individuals with LS increased significantly with advancing age among both men and women (Fig. 1,* P* < 0.001). Further details on LS classification based on the two-step test, stand-up test, and GLFS-25 are provided in Supplementary Table S3.

### Multivariate analysis of the effect of MetS on LS

Overall, LS was observed in 23.6% (LS = 622; non-LS = 2018) of the MetS-positive participants and 14.3% (LS = 4643; non-LS = 27,776) of the MetS-negative participants (*P* < 0.001). For logistic regression analysis, participants with LS (LS_1–3) were classified as “LS,” and models were constructed including MetS, sex, and age. As shown in Table 2, MetS was identified as a risk factor for LS (odds ratio [OR] 1.82 [1.65–2.01], *P* < 0.0001). Women were at a higher risk of LS than men (OR 1.59 [1.49–1.68], *P* < 0.0001). The risk of LS increased with age, and the OR was the highest among the participants in their 60 s and > 70 years of age, followed by those in their 40 s and 50 s.

**Table 2:** Logistic regression analysis for data of participants with locomotive syndrome.

	OR (95% CI)	*P*-value
Metabolic syndrome	1.82 (1.65–2.01)	< 0.001
Sex (Women/Men)^†^	1.59 (1.49–1.68)	< 0.001
Age, years^‡^		
> 70/40–49	3.95 (3.42–4.57)	< 0.001
60–69/40–49	1.86 (1.70–2.03)	< 0.001
50–59/40–49	1.51 (1.40–1.62)	< 0.001
< 39/40–49	0.75 (0.67–0.83)	< 0.001

Based on the results of the Locomotor test, all of LS_1–LS_3 were collectively defined as test positive.

*OR* odds ratio, *CI* confidence interval.

^†^Reference group: Men.

^‡^Reference group: Age 40–49 years.

**Fig. 2:**
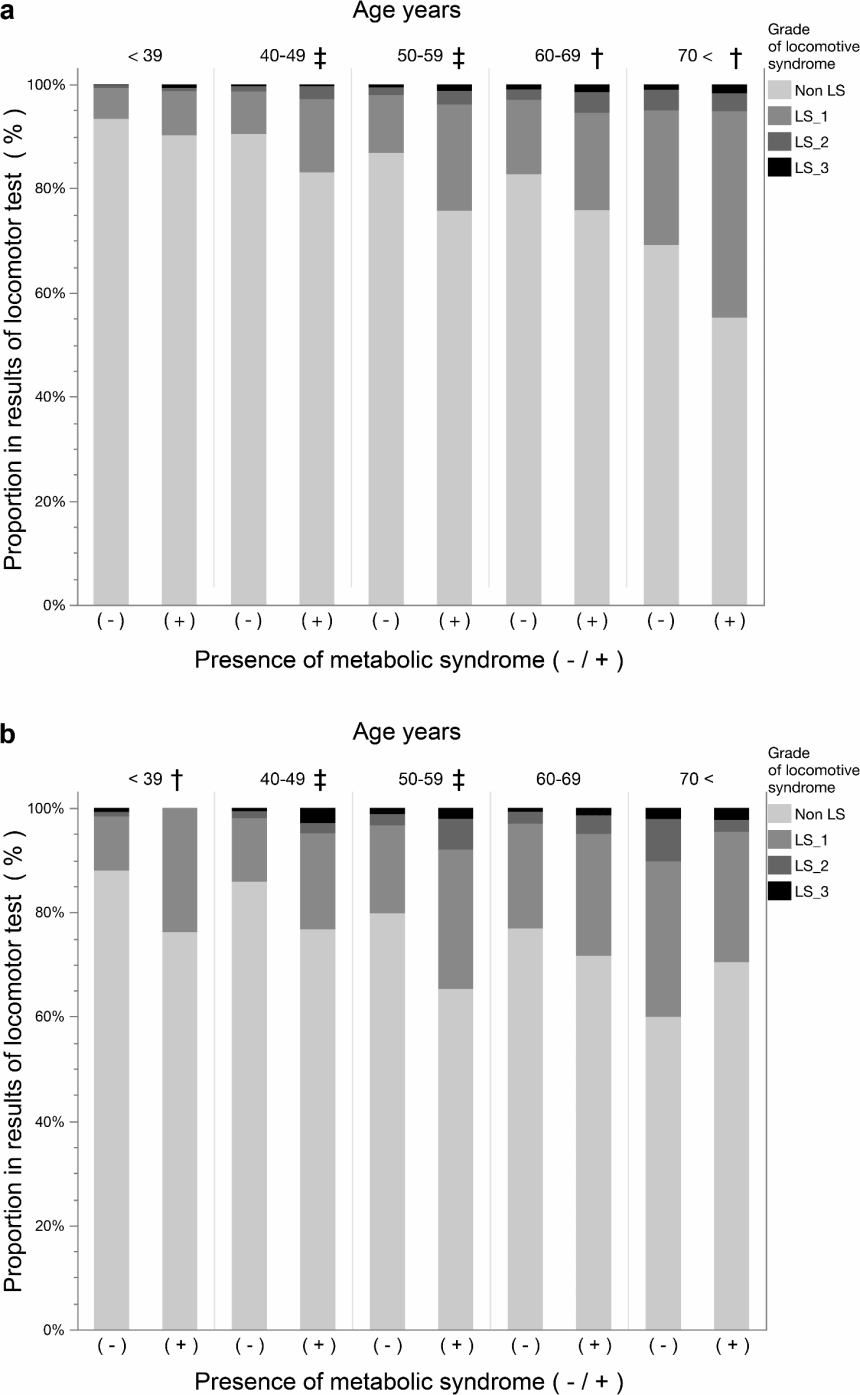
Distribution of locomotor test outcomes by the presence of metabolic syndrome by age group. (**A**) This bar graph shows the proportions of the results of the locomotive syndrome risk test for participants categorized by age group for men with and without metabolic syndrome. Proportions were tested using the chi-square test for each age group. (**B**) This panel shows the proportions of the results of the locomotive syndrome risk test for participants categorized by age group for women with and without metabolic syndrome. Proportions were tested using the chi-square test for each age group. †*P* < 0.05, ‡*P* < 0.001.

The relationship between the presence or absence of MetS and the distribution of LS stage locomotor test outcomes was categorized by sex and age group (Fig. 2A,B). Among men, the proportion of LS-positive participants was significantly higher in the MetS group for those in their 40 s, 50 s, 60 s, and 70 s (*P* < 0.05). Among women, LS prevalence was significantly higher in the MetS group for those in their 30 s, 40 s, and 50 s (*P* < 0.05). However, among women in their 70 s, LS-positive participants were significantly more prevalent in the non-MetS group. The prevalence of LS among individuals with and without MetS by age group, along with the corresponding ratios, is also shown in Supplementary Table S3. The prevalence ratio of LS in participants in their 50 s was 2.11 for men and 2.10 for women, representing the highest value among men and the second highest among women. The highest ratio among women was observed in participants under 39 years of age, at 2.29 (Supplementary Table S4).

### Clinical characteristics of participants with LS and MetS and the associated complications

Men and women in their 50 s (M = 6791; W = 4683) were selected for subgroup analysis because this age group showed the highest prevalence of LS among male participants with MetS and the second highest among female participants. Participants were categorized into subgroups based on their LS and MetS status (Table 3). The groups were divided into the healthy group (-/-), the LS alone group (-/LS), the MetS alone group (MetS/-), and the combined MetS and LS group (MetS/LS). The proportion of participants in each group was in the order -/LS, MetS/-, and MetS/LS, with 11.6%, 8.7%, and 2.8% for men and 18.9%, 4.0%, and 2.1% for women, respectively. Comparison of clinical data showed significantly higher abdominal circumference and blood glucose levels in the MetS/LS group than those in the other groups in both men and women (*P* < 0.001). When comparing treatment histories, a greater proportion of participants in the MetS/LS group received treatment for all three conditions: antihypertensive medication, diabetes treatment, and lipid metabolic disorders, compared to the overall groups.

**Table 3:** Clinical characteristics of participants in their 50 s with/without locomotive syndrome and metabolic syndrome.

Sex (n)	Men (6791)					Women (4683)				
MetS (n)	Without MetS (6012)		With MetS (779)		*P*-value	Without MetS (4395)		With MetS (288)		*P*-value
LS	Non-LS	LS	Non-LS	LS		Non-LS	LS	Non-LS	LS	
n (%)	5221 (76.9)	791 (11.6)	590 (8.7)	189 (2.8)		3508 (74.9)	887 (18.9)	188 (4.0)	100 (2.1)	
Age (mean ± SD)	54.1 ± 2.9	54.4 ± 2.9	54.1 ± 2.8	54.4 ± 2.8		54.1 ± 2.8	54.2 ± 2.8	54.2 ± 2.9	54.8 ± 2.7	
Waist circumference (cm, mean ± SD)	82.0 ± 6.9	84.6 ± 9.8	93.5 ± 7.7	97.3 ± 8.9	*	75.5 ± 8.0	79.5 ± 9.8	88.0 ± 8.0	92.7 ± 9.5	*
Systolic BP (mmHg, mean ± SD)	119.4 ± 14.2	119.3 ± 14.7	134.2 ± 15.2	134.2 ± 15.7		114.6 ± 15.3	116.4 ± 15.1	133.6 ± 16.8	131.9 ± 15.2	
Diastolic BP (mmHg, mean ± SD)	77.7 ± 10.8	77.3 ± 11.1	88.2 ± 10.8	87.1 ± 11.1		70.4 ± 11.6	71.1 ± 11.5	82.6 ± 11.8	80.9 ± 10.6	
Glucose (mg/dL, mean ± SD)	101.3 ± 12.9	103.3 ± 18.6	119.7 ± 25.2	127.8 ± 40.1	*	95.0 ± 9.6	96.3 ± 12.2	110.0 ± 16.0	120.0 ± 31.6	*
HDL (mg/dL, mean ± SD)	58.1 ± 13.7	46.2 ± 10.4	45.9 ± 10.4	45.9 ± 10.4		73.2 ± 15.6	70.5 ± 15.2	51.3 ± 11.1	52.1 ± 13.5	
TG (mg/dL, mean ± SD)	103.9 ± 61.1	106.2 ± 53.1	197.9 ± 113.6	204.2 ± 108.4		73.6 ± 36.2	82.0 ± 60.4	169.9 ± 103.7	1158.9 ± 59.1	
Tx for hypertension (n, %)	966 (18.5)	200 (25.3)	233 (39.5)	87 (46.0)	†	314 (9.0)	102 (11.5)	56 (28.9)	39 (39.0)	†
Tx for type 2 diabetes (n, %)	243 (4.7)	71 (9.0)	99 (16.8)	47 (24.9)	†	34 (1.0)	19 (2.1)	15 (8.0)	22 (22.0)	†
Tx for hyperlipidemia (n, %)	728 (8.3)	135 (8.0)	143 (18.4)	63 (21.8)	†	337 (3.9)	107 (6.4)	38 (4.9)	38 (13.1)	†

*BP* blood pressure, *HDL* high-density lipoprotein, *LS* locomotive syndrome, *MetS* metabolic syndrome, *Tx* treatment.

^†^Significant difference in chi-square test (*P* < 0.001).

*A significant difference was found between the groups with MetS and LS and the other groups by the Dunnet test (*P* < 0.01).

## Discussion

This study analyzed real-world data from physical function assessments conducted during health check-ups ranging from middle to late age. We elucidated the distribution of locomotive disorders in adults aged 30–70 years and demonstrated that MetS was associated with LS and served as a risk factor. Patients with MetS exhibited a higher prevalence of LS across all age categories, particularly in their 50 s. Individuals in this age range with MetS were more likely to have obesity and glucose tolerance abnormalities, and a greater proportion were receiving treatment for hypertension, hyperlipidemia, and diabetes than those without the syndrome. This study emphasizes the importance of health examinations, including exercise stress tests in midlife, and suggests that they are effective in screening for LS and identifying individuals at risk of mobility impairment, particularly among patients with MetS^24,25^.

In a world where the aging population is continually growing, societal efforts to maintain the health of middle-aged and older adults are crucial^26^. The association between LS, a risk factor for disability, and MetS, a major public health issue, need to be elucidated urgently^27^. Several intervention trials have been conducted to evaluate the risk of disability in older people, which have been suggested as useful in preventing frailty by identifying and intervening with high-risk older people^28,29^. However, this report is the only example of these evaluation methodologies being socially implemented and executed, and there is no other large-scale real-world data analysis of integrated physical function evaluations and health check-up data involving over 35,000 individuals. This suggests that the LS examination is an effective assessment tool for evaluating the risk of age-related frailty, demonstrating its broad applicability for implementation within social and healthcare systems.

This study found that 7.7% and 18.1% of middle-aged men and women, respectively, who underwent physical examinations had decreased mobility, which increased with age. This is consistent with a previous observational study, which reported that LS began in middle age and increased with age^30^, which is a consequence of reduced lower limb mobility in healthy middle-aged people^31,32^. LS tests correlate with impaired maximal walking speed and lower limb muscle strength, although these signs are also found in patients with metabolic abnormalities, such as impaired glucose tolerance^33,34^. Therefore, the correlation with MetS was analyzed to clarify the characteristics of this midlife-onset frailty.

Therefore, we selected individuals in their 50 s for subgroup analysis. This age group had the highest prevalence ratio of LS among men and the second highest among women. Furthermore, the absolute number of individuals with both LS and MetS was the largest in this age group, supporting its relevance for more detailed stratified analysis. Previous studies have investigated the relationship between MetS and mobility impairment in older populations, including cohort studies analyzing MetS as a risk factor for disability in older adults and small-sample studies examining the coexistence of MetS and LS in older individuals^35–37^. However, our study provides novel insights by analyzing a significantly larger dataset (35,059 individuals) and focusing specifically on middle-aged adults, highlighting the importance of early intervention before substantial musculoskeletal decline occurs.

The observed sex differences in MetS and LS prevalence may be explained by differences in muscle mass and visceral fat accumulation between men and women. Men generally have greater muscle mass and higher visceral fat accumulation, whereas women tend to have lower levels of both^38,39^. These differences likely contribute to the distinct patterns of LS development, where central obesity plays a larger role in LS development among men, while muscle mass decline is the predominant factor in women. As a result, LS prevalence remained consistently higher in the MetS group among men across all age groups. However, in women, the proportion of LS-positive individuals in the non-MetS group increased with age, surpassing that of the MetS group in older adults. This suggests that age-related muscle loss becomes the dominant risk factor for LS in women, exceeding the influence of MetS in later life^40^.

Considering the interplay between metabolic and musculoskeletal factors, the transition from middle to older age is a critical period for LS development. Changes in lipid metabolism and muscle mass have been reported to follow a nonlinear trajectory with age, particularly around the ages of 44 and 60^41^, indicating that the coexistence of MetS and LS in the 50 s may reflect an accelerated aging process. This highlights the importance of evaluating both conditions during routine health check-ups to facilitate early intervention. From a preventive medicine perspective, recognizing the presence of MetS and LS comorbidity in middle-aged individuals is particularly relevant, as it allows for targeted interventions such as lifestyle modifications and exercise programs before irreversible musculoskeletal decline occurs.

Furthermore, patients with an overlap between MetS and LS were more likely to be obese, have impaired glucose tolerance, and have previously been diagnosed and treated for hypertension, diabetes, and lipid metabolic disorders, than the non-comorbid group. Compared with those without MetS, individuals with LS had larger waist circumferences and a higher prevalence of hypertension. Previous reports have indicated that the combination of locomotor disorders and MetS in older people increases the risk of central adiposity, high triglyceride levels, and elevated fasting glucose^36,37^. Furthermore, diabetes has been linked to poor lower limb motor balance^41–44^. Thus, we envisage that numerous health conditions associated with obesity would be able to be diagnosed at an early stage, while also assessing MetS and LS.

The results of this study suggest that a combined assessment of LS and MetS during regular health check-ups in the middle to old-age period may allow for the early detection of patients at a high risk of developing frailty and metabolic disorders. We believe that MetS and LS share common biological mechanisms, and the coexistence of the two syndromes accelerates the decline in mobility. In sarcopenic obesity, aging and visceral fat accumulation trigger an increase in inflammatory cytokines and oxidative stress, which promote muscle loss and mitochondrial dysfunction^45,46^. A similar mechanism is thought to occur in individuals with both MetS and LS. Therefore, we consider the coexistence of MetS and LS as a pre-sarcopenic obesity state, and assessing both conditions during health check-ups can be effective for the early detection and prevention of sarcopenic obesity. It would, therefore, be feasible to provide preventive interventions for identified high-risk patients. However, because this is a cross-sectional study, the causal association between LS and MetS remains unknown.

This analysis did not collect information on participants’ lifestyles or exercise habits, leaving uncertainties regarding the preventive measures that should be prioritized. To comprehensively evaluate the long-term impact of MetS and LS and to develop effective prevention strategies, further longitudinal studies incorporating more detailed lifestyle and behavioral data are needed.

Additionally, the data used in this study were collected during the late phase of the SARS-CoV-2 pandemic, and its potential impact on lifestyle behaviors cannot be ruled out. However, despite some remaining behavioral restrictions, many individuals had resumed their daily lives, as evidenced by the significant number of participants who continued to undergo health check-ups as usual. Because this study is based on a single-year dataset from Ningen Dock, the overall societal impact of the pandemic may exist, but its influence on the association between MetS and LS is considered minimal. To further assess the long-term effects of the pandemic on this association, longitudinal studies using extended datasets would be valuable. In particular, to clarify the specific influence of lifestyle on MetS and LS, a longitudinal analysis using data from 2018 to 2022 would be essential.

This study did not collect specific data on participants’ occupations, income levels, or on urban/rural distribution. However, as Omiya City Clinic is located in Saitama City, it is reasonable to assume that most participants resided in this urban area. According to the 2020 Census, Saitama City has a population of 1.32 million, with a labor force of 615,000^47^. Our sample represents nearly 5% of this working-age population, suggesting that the findings reflect urban workers in Japan.

As the health check-up was self-funded, participants likely had a certain degree of financial stability, predominantly belonging to middle to upper-middle income groups. While this study provides insights into MetS and LS distribution among urban workers, its findings may not be fully generalizable to other populations. Future studies should include more diverse samples and incorporate objective measurements to enhance reliability.

In conclusion, this study demonstrated the efficacy of monitoring the relationship between MetS and LS beginning in midlife and emphasized the need for early intervention as a preventive approach. We re-evaluated the role of health examinations in supporting functional independence and improving quality of life, opening possibilities for a comprehensive public health policy.

## Electronic supplementary material

Below is the link to the electronic supplementary material.

Supplementary Material 1

## Data Availability

The data analyzed in this study were obtained from individuals who underwent health check-ups at the Omiya City Clinic. The data were anonymized before analysis. As the data originate from a single clinical site and are not open access, they are not publicly available. Researchers seeking access to the data may contact the corresponding author for inquiries regarding data sharing and collaboration opportunities.
