# Supplementary material for: Prevalence and coexistence of locomotive syndrome with reduced mobility and metabolic syndrome: a cross-sectional study of 35,059 Japanese adults
Source: Sci Rep. 2025 Apr 19;15:13547. doi: 10.1038/s41598-025-98288-2 (PMC12009296; doi:10.1038/s41598-025-98288-2)
Supplement: Supplementary file 1 — Supplementary Material 1 [file 41598_2025_98288_MOESM1_ESM.docx]

**Supplementary information**

**Prevalence and coexistence of locomotive syndrome with reduced mobility and metabolic syndrome: A cross-sectional study of 35,059 Japanese adults**

Chihiro Goto^1,2,3^, Kohei Maruya^4^, Yasuhiro Morita^5^, Tomoyuki Arai^5^, Satoshi Yamaguchi^6^, Keiko Yamada^7,8^, Masaru Moriyama^3^, Hideaki Ishibashi^9^, Ryo Nakagawa^1,2,3,*^.

^1^Division of Advanced Preventive Medicine, Chiba University, 1-8-1 Inohana, Chuo-ku, Chiba 260-8670, Japan

^2^Department of Gastroenterology, Graduate School of Medicine, Chiba University, 1-8-1 Inohana, Chuo-ku, Chiba 260-8670, Japan

^3^Omiya City Clinic, 1-7-5 Sakuragicho, Omiya-ku, Saitama City, Saitama 330-8669, Japan

^4^Department of Physical Therapy, School of Health Sciences, Japan University of Health Science, 2-555, Suga, Satte, Saitama 340-0145, Japan

^5^Department of Physical Therapy Faculty of Health and Medical Care, Saitama Medical University, 981, Kawakado, Iruma, Saitama 350-0436, Japan

^6^Graduate School of Global and Transdisciplinary Studies, Chiba University, 1-8-1 Inohana, Chuo-ku, Chiba 260-8670, Japan

^7^Department of Liberal Arts, Faculty of healthcare and welfare, Saitama Prefectural University, 820 Sannomiya, Koshigaya-shi, Saitama 343-8540, Japan

^8^Department of Rehabilitation, the University of Tokyo Hospital 7-3-1, Hongo, Bunkyo-ku, Tokyo, 113-0033, Japan

^9^Department of Orthopedic surgery, INA hospital, 5014-1 Komuro, Kitaadachi-Ina, Saitama 362-0806, Japan

^*^**Corresponding author**

Ryo Nakagawa, M.D., PhD.

Division of Advanced Preventive Medicine, Chiba University, 1-8-1 Inohana, Chuo-ku, Chiba 260-8670, Japan

Telephone: +81-43-226-2083, Fax: +81-43-226-2088

E-mail: [ryo420@chiba-u.jp](mailto:ryo420@chiba-u.jp)

**Supplementary Table S1.** Comprehensive grading method for the locomotive syndrome risk test.

| **Grade of the risk for locomotive syndrome** | **Stand-Up test** | **Two-Step test** | **25-GLSF** |
| --- | --- | --- | --- |
| LS_1 | Fail at one-leg/40 cm | < 1.3 | ≥ 7 points |
| LS_2 | Fail at both-legs/20 cm | < 1.1 | ≥ 16 points |
| LS_3 | Fail at both-legs/30 cm | < 0.9 | ≥ 24 points |

The overall risk grade for locomotive syndrome is the highest risk grade among the evaluation of the three tests, “Stand-Up test,” “Two-Step test,” and “25-question geriatric locomotive function scale.”

25-GLSF, 25-question geriatric locomotive function scale.

**Supplementary Table S2.** Rating table for translated 25-question geriatric locomotive function scale.

| **The 25-question risk assessment** | | | | | | | | |
| --- | --- | --- | --- | --- | --- | --- | --- | --- |
| - **We are inquiring about any pain or discomfort you have experienced in the last month.** | | | | | | | | |
| Q1. Have you felt any pain or numbness in your neck, shoulders, arms, or hands? | | | | | | | | |
| A. | Not at all | |  |  | | |  | |
| B. | A little | |  |  | | |  | |
| C. | Moderately | |  |  | | |  | |
| D. | Quite a bit | |  |  | | |  | |
| E. | Extremely | |  |  | | |  | |
|  |  | |  |  | | |  | |
| Q2. Have you experienced pain in your back, lower back, or buttocks? | | | | | | | | |
| A. | Not at all | |  |  | | |  | |
| B. | A little | |  |  | | |  | |
| C. | Moderately | |  |  | | |  | |
| D. | Quite a bit | |  |  | | |  | |
| E. | Extremely | |  |  | | |  | |
|  |  | |  |  | | |  | |
| Q3. Have you felt any pain or numbness in any part of your lower limbs, including hips, thighs, knees, calves, shins, ankles, or feet? | | | | | | | | |
| A. | Not at all | |  |  | | |  | |
| B. | A little | |  |  | | |  | |
| C. | Moderately | |  |  | | |  | |
| D. | Quite a bit | |  |  | | |  | |
| E. | Extremely | |  |  | | |  | |
|  |  | |  |  | | |  | |
| Q4. How difficult is it for you to move your body during everyday activities? | | | | | | | | |
| A. | Not at all | |  |  | | |  | |
| B. | A little | |  |  | | |  | |
| C. | Moderately | |  |  | | |  | |
| D. | Quite a bit | |  |  | | |  | |
| E. | Extremely | |  |  | | |  | |
|  |  | |  |  | | |  | |
| - **We would also like to know about your daily activities over the last month.** | | | | | | | | |
| Q5. How difficult is it for you to get up from or lie down in bed? | | | | | | | | |
| A. | Not at all | |  |  | | |  | |
| B. | A little | |  |  | | |  | |
| C. | Moderately | |  |  | | |  | |
| D. | Quite a bit | |  |  | | |  | |
| E. | Extremely | |  |  | | |  | |
|  |  | |  |  | | |  | |
| Q6. How much difficulty do you have rising from a seated position? | | | | | | | | |
| A. | Not at all | |  |  | | |  | |
| B. | A little | |  |  | | |  | |
| C. | Moderately | |  |  | | |  | |
| D. | Quite a bit | |  |  | | |  | |
| E. | Extremely | |  |  | | |  | |
|  |  | |  |  | | |  | |
| Q7. How difficult is it for you to walk around inside your home? | | | | | | | | |
| A. | Not at all | |  |  | | |  | |
| B. | A little | |  |  | | |  | |
| C. | Moderately | |  |  | | |  | |
| D. | Quite a bit | |  |  | | |  | |
| E. | Extremely | |  |  | | |  | |
|  |  | |  |  | | |  | |
| Q8. How challenging is it for you to put on or take off a shirt? | | | | | | | | |
| A. | Not at all | |  |  | | |  | |
| B. | A little | |  |  | | |  | |
| C. | Moderately | |  |  | | |  | |
| D. | Quite a bit | |  |  | | |  | |
| E. | Extremely | |  |  | | |  | |
|  |  | |  |  | | |  | |
| Q9. How much difficulty do you have putting on or taking off trousers or pants? | | | | | | | | |
| A. | Not at all | |  |  | | |  | |
| B. | A little | |  |  | | |  | |
| C. | Moderately | |  |  | | |  | |
| D. | Quite a bit | |  |  | | |  | |
| E. | Extremely | |  |  | | |  | |
|  |  | |  |  | | |  | |
| Q10. How difficult is it for you to manage your personal toileting needs? | | | | | | | | |
| A. | Not at all | |  |  | | |  | |
| B. | A little | |  |  | | |  | |
| C. | Moderately | |  |  | | |  | |
| D. | Quite a bit | |  |  | | |  | |
| E. | Extremely | |  |  | | |  | |
|  |  | |  |  | | |  | |
| Q11. How hard is it for you to bathe and wash yourself? | | | | | | | | |
| A. | Not at all | |  |  | | |  | |
| B. | A little | |  |  | | |  | |
| C. | Moderately | |  |  | | |  | |
| D. | Quite a bit | |  |  | | |  | |
| E. | Extremely | |  |  | | |  | |
|  |  | |  |  | | |  | |
| Q12. How challenging is it for you to climb or descend stairs? | | | | | | | | |
| A. | Not at all | |  |  | | |  | |
| B. | A little | |  |  | | |  | |
| C. | Moderately | |  |  | | |  | |
| D. | Quite a bit | |  |  | | |  | |
| E. | Extremely | |  |  | | |  | |
|  |  | |  |  | | |  | |
| Q13. How difficult is it for you to walk quickly? | | | | | | | | |
| A. | Not at all | |  |  | | |  | |
| B. | A little | |  |  | | |  | |
| C. | Moderately | |  |  | | |  | |
| D. | Quite a bit | |  |  | | |  | |
| E. | Extremely | |  |  | | |  | |
|  |  | |  |  | | |  | |
| Q14. How difficult do you find it to groom yourself before going out? | | | | | | | | |
| A. | Not at all | |  |  | | |  | |
| B. | A little | |  |  | | |  | |
| C. | Moderately | |  |  | | |  | |
| D. | Quite a bit | |  |  | | |  | |
| E. | Extremely | |  |  | | |  | |
| Q15. How much distance can you walk comfortably without halting? (Please choose the closest answer) | | | | | | | | |
| A. | More than 2–3 km | | |  | | |  | |
| C. | About 300 m | | |  | | |  | |
| D. | About 100 m | | |  | | |  | |
| E. | About 10 m | | |  | | |  | |
|  |  | |  | | |  | |  |
| Q16. How much difficulty do you face when going out to local places? | | | | | | | | |
| A. | Not at all | |  |  | | |  | |
| B. | A little | |  |  | | |  | |
| C. | Moderately | |  |  | | |  | |
| D. | Quite a bit | |  |  | | |  | |
| E. | Extremely | |  |  | | |  | |
|  |  | |  |  | | |  | |
| Q17. How burdensome is it for you to carry home around 2 kg of shopping items, such as two 1-liter packs of milk? | | | | | | | | |
| A. | Not at all | |  |  | | |  | |
| B. | A little | |  |  | | |  | |
| C. | Moderately | |  |  | | |  | |
| D. | Quite a bit | |  |  | | |  | |
| E. | Extremely | |  |  | | |  | |
|  |  | |  |  | | |  | |
| Q18. How difficult is it for you to use public transportation, like trains or buses? | | | | | | | | |
| A. | Not at all | |  |  | | |  | |
| B. | A little | |  |  | | |  | |
| C. | Moderately | |  |  | | |  | |
| D. | Quite a bit | |  |  | | |  | |
| E. | Extremely | |  |  | | |  | |
|  |  | |  |  | | |  | |
| Q19. How challenging is light housework for you, such as preparing meals, cleaning up, or tidying? | | | | | | | | |
| A. | Not at all | |  |  | | |  | |
| B. | A little | |  |  | | |  | |
| C. | Moderately | |  |  | | |  | |
| D. | Quite a bit | |  |  | | |  | |
| E. | Extremely | |  |  | | |  | |
|  |  | |  |  | | |  | |
| Q20. How tough is it for you to perform heavy household tasks, like using a vacuum cleaner or managing bedding? | | | | | | | | |
| A. | Not at all | |  |  | | |  | |
| B. | A little | |  |  | | |  | |
| C. | Moderately | |  |  | | |  | |
| D. | Quite a bit | |  |  | | |  | |
| E. | Extremely | |  |  | | |  | |
|  |  | |  |  | | |  | |
| Q21. How hard is it for you to participate in physical activities like sports or dancing? | | | | | | | | |
| A. | Not at all | |  |  | | |  | |
| B. | A little | |  |  | | |  | |
| C. | Moderately | |  |  | | |  | |
| D. | Quite a bit | |  |  | | |  | |
| E. | Extremely | |  |  | | |  | |
|  |  | |  |  | | |  | |
| Q22. Are you limiting your social interactions with close friends and acquaintances? | | | | | | | | |
| A. | Not at all | |  |  | | |  | |
| B. | A little | |  |  | | |  | |
| C. | Moderately | |  |  | | |  | |
| D. | Quite a bit | |  |  | | |  | |
| E. | Completely | |  |  | | |  | |
|  |  | |  |  | | |  | |
| Q23. Are you refraining yourself from participating in local community events or activities? | | | | | | | | |
| A. | Not at all | |  |  | | |  | |
| B. | A little | |  |  | | |  | |
| C. | Moderately | |  |  | | |  | |
| D. | Quite a bit | |  |  | | |  | |
| E. | Completely | |  |  | | |  | |
|  |  | |  |  | | |  | |
| Q24. How frequently do you feel anxious about the chance of falling in your home? | | | | | | | | |
| A. | Not at all | |  |  | | |  | |
| B. | A little | |  |  | | |  | |
| C. | Moderately | |  |  | | |  | |
| D. | Quite a bit | |  |  | | |  | |
| E. | Extremely | |  |  | | |  | |
|  |  | |  |  | | |  | |
| Q25. How concerned are you about the prospect of being unable to walk in the future? | | | | | | | | |
| A. | Not at all | |  |  | | |  | |
| B. | A little | |  |  | | |  | |
| C. | Moderately | |  |  | | |  | |
| D. | Quite a bit | |  |  | | |  | |
| E. | Extremely | |  |  | | |  | |
| **Response Tally and Score Calculation:** | | | | | | |  | |
| **Response** | **Count** | | **Points per Response** | | | | **Subtotal Score** | |
| A |  | | 0 | | | |  | |
| B |  | | 1 | | | |  | |
| C |  | | 2 | | | |  | |
| D |  | | 3 | | | |  | |
| E |  | | 4 | | | |  | |
| Total | 25 | | **Total Score** | | | |  | |
| **Overall rating of the 25-question risk assessment** | | | | | | | | |
| **Total Score Range** | | **LS Level** | | | **Condition Description** | | | |
| 7–less than 16 | | LS_1 | | | Beginning of decline in mobility function. | | | |
| 16–less than 24 | | LS_2 | | | Progressive decline in mobility function. | | | |
| 24 and above | | LS_3 | | | Advanced decline in mobility function, affecting social participation. | | | |

This presents a translated version of the 25-question risk assessment. This table is a translation of the assessment method presented in Japanese on the official website of the Japanese Orthopedic Association (https://locomo-joa.jp/check/test/locomo25).

**Supplementary Table S3. Three tests used for LS classification: Two-step test, Stand-up test, and GLFS-25.**

| **Two-step test** | **LS grade** | **Men** | **Women** | ***** |
| --- | --- | --- | --- | --- |
| ≥ 1.3 | Non-LS | 20,327 (97.37%) | 13,547 (95.51%) |  |
| < 1.3 | LS_1 | 516 (2.47%) | 623 (4.39%) |  |
| < 1.1 | LS_2 | 32 (0.15%) | 14 (0.10%) |  |
| < 0.9 | LS_3 | 0 (0 %) | 0 (0 %) |  |
| **Stand-up test** |  |  |  | * |
| Succeed at one-leg / 40 cm | Non-LS | 20,499 (98.20%) | 13,703 (96.61%) |  |
| Fail at one-leg / 40 cm | LS_1 | 336 (1.61%) | 453 (3.19%) |  |
| Fail at both-legs / 20 cm | LS_2 | 36 (0.17%) | 26 (0.18%) |  |
| Fail at both-legs / 30 cm | LS_3 | 4 (0.02%) | 2 (0.01%) |  |
| **GLFS-25** |  |  |  | * |
| ≥ 6 points | Non-LS | 18,656 (89.37%) | 12,154 (85.69%) |  |
| ≥ 7 points | LS_1 | 1,850 8.86% | 1,658 (11.69%) |  |
| ≥ 16 points | LS_2 | 254 (1.22%) | 243 (1.71%) |  |
| ≥ 24 points | LS_3 | 115 (0.55%) | 129 (0.91%) |  |

This table presents the distribution of LS classification based on three different assessments: the two-step test, stand-up test, and GLFS-25. The final LS classification was determined using the lowest (worst) performance among these three tests. LS severity was categorized into four stages: Non-LS (normal mobility), LS_1 (onset of reduced mobility), LS_2 (progression in mobility decline), and LS_3 (advanced stage with impaired social participation). Percentages indicate the proportion within each sex. Significant differences between men and women were assessed using the chi-square test (*p < 0.05).*

**Supplementary Table S4.** Prevalence of locomotive syndrome by age group of participants with and without metabolic syndrome

|  | **Metabolic syndrome** | | **Ratio** |
| --- | --- | --- | --- |
|  | **with** | **without** |  |
| **Men** | % (LS/non-LS) | % (LS/non-LS) | with (%) /without (%) |
| <39 | 10.9% (15/138) | 7.1% (183/2,574) | 1.53 |
| 40–49 | 20.4% (102/500) | 10.5% (647/6,138) | 1.94 |
| 50–59 | 32.0% (189/590) | 15.2% (791/5221) | 2.11 |
| 60–69 | 31.9% (79/248) | 20.9% (488/2,334) | 1.52 |
| >70 | 81.3% (26/32) | 44.6% (179/401) | 1.82 |
| **Women** | % (LS/non-LS) | % (LS/non-LS) | with (%) /without (%) |
| <39 | 31.3% (10/32) | 13.6% (250/1,833) | 2.29 |
| 40–49 | 30.4% (48/158) | 16.5% (699/4,246) | 1.85 |
| 50–59 | 53.2% (100/188) | 25.3% (887/3,508) | 2.10 |
| 60–69 | 39.6% (40/101) | 30.0% (406/1,352) | 1.32 |
| >70 | 41.9% (13/31) | 66.9% (113/169) | 0.63 |

Participants were classified according to their sex, age, and whether they had metabolic syndrome. The table illustrates the prevalence of locomotive syndrome, consolidated across all severity levels, within each group. To understand the impact of metabolic syndrome, the prevalence of locomotive syndrome in individuals with metabolic syndrome was expressed as a ratio relative to its prevalence in those without metabolic syndrome, further segmented by sex and age.
